# Maintenance of an Intraoperative External Fixator Is Associated With Decreased Postoperative Stiffness After Definitive Management of High-Energy Tibial Plateau Fractures

**DOI:** 10.7759/cureus.38561

**Published:** 2023-05-04

**Authors:** Jacob Speybroeck, Tyler Moon, Lucas Haase, Douglas Haase, Brent Wise, George Ochenjele, Joshua Napora

**Affiliations:** 1 Department of Orthopaedic Surgery, University Hospitals Cleveland Medical Center, Cleveland, USA; 2 Department of Orthopaedic Surgery, University of Kansas School of Medicine, Kansas City, USA

**Keywords:** arthrofibrosis, stiffness, open reduction internal fixation, external fixator, tibial plateau fractures

## Abstract

Introduction

Postoperative stiffness is a common complication after high-energy tibial plateau fractures. Investigation into reported surgical techniques for the prevention of postoperative stiffness is limited. The purpose of this study was to compare the rates of postoperative stiffness after second-stage definitive surgery for high-energy tibial plateau fractures between groups of patients who had the external fixator prepped into the surgical field and those who did not.

Methods

Two hundred forty-four patients met the inclusion criteria between the two academic Level I trauma centers, representing the retrospective observational cohort. Patients were separated based on prepping of the external fixator into the surgical field during second-stage definitive open reduction and internal fixation. One hundred sixty-two patients were in the prepped group and 82 were in the non-prepped group. Post-operative stiffness was determined by the need to return to the operating room for subsequent procedures.

Results

At the final follow-up (mean = 14.6 months), patients in the non-prepped group had an increased rate of stiffness post-operatively (18.3% non-prepped versus 6.8% prepped; p = 0.006). No other investigated variables were associated with increased post-operative stiffness, including the number of days spent in the fixator and operative time. The relative risk for post-operative stiffness associated with complete fixator removal was 2.54 (95% CI 1.26-4.41; p = 0.008 on binary logistic regression; absolute risk reduction 11.5%).

Conclusion

At the final follow-up, maintenance of an intraoperative external fixator as a reduction aid was associated with a clinically significant decrease in post-operative stiffness after definitive management of high-energy tibial plateau fractures, when compared with complete removal prior to prepping.

## Introduction

High-energy tibial plateau fractures are frequently treated with staged management, allowing for soft tissue rest, hoping to mitigate complications. While two-stage management of these more severe injuries is considered the standard, there is little consensus as it pertains to the management of the external fixator at the time of definitive fixation [1]. A survey of 50 surgeons frequently treating closed fractures of extremities demonstrated that 86% of surgeons prefer the complete removal of the external fixator prior to sterile preparation and draping [2]. Multiple studies have shown that prepping the external fixator for tibial plateau fractures is safe and does not infer an additional infection risk [3,4]. However, the benefits, if any, of sterilely including the external fixator in the surgical field on postoperative outcomes are not well documented. This technique is thought to aid in extremity control in positioning and assist fracture reduction. In addition, many utilize the distraction and ligamentotaxis gained from the femoral distractor in treating these fractures [5].

High-energy fractures of the tibial plateau are commonly associated with significant patient morbidity. One such complication is knee stiffness, which has been reported in up to 15% of these injuries and often requires additional intervention [1,6,7,8]. Recent investigations have identified non-white ethnicity, increasing age, deep infection, bilateral injuries, and time in external fixator devices as risk factors for this development [6,9,10]. To date, there is limited published literature on the intra- and post-operative benefits of maintaining the external fixator during the definitive surgical procedure, including its impact on postoperative stiffness. This study aims to determine if prepping the external fixator has an impact on the rates of postoperative knee stiffness after staged definitive fixation of a high-energy tibial plateau fracture.

## Materials and methods

After institutional review board approval, a retrospective cohort study was conducted at two academic level one trauma centers. This study required no additional sources of funding. Patients were identified by a database CPT code search, including all patients who underwent open reduction internal fixation of high-energy tibial plateau fractures between 2015 and 2019. High-energy tibial plateau fractures were defined as an injury treated with staged management, and application of an external fixator followed by delayed definitive fixation. Exclusion criteria were patients less than 18 years of age, treatment with a hexapod frame, other ipsilateral fractures of the tibia, less than 3 months follow-up, or deficient demographic, surgical, or postoperative records. Patients were typically evaluated for follow-up at 2-week, 6-week, and 12-week periods, and then at the discretion of the operating surgeon.

Patient data including age, gender, body mass index (BMI), Orthopedic Trauma Association (OTA/AO) fracture classification, open fractures, compartment syndrome diagnosis, and days in external fixation were collected from inpatient electronic records. Fractures were classified with plain knee radiographs (anterior/posterior and lateral) prior to surgical intervention. All included patients were treated in a staged manner, with the application of an external fixator followed by delayed definitive fixation when the operating surgeon deemed the soft tissue was amenable. Definitive fixation was performed by or under the direction of a fellowship-trained orthopedic traumatologist. All external fixators included in the study were trans-articular or knee-spanning, with pins placed in the tibia and femur. All external fixators were removed after definitive fixation.

The data was gathered for sterile preparation methods from operative reports for the definitive procedure. Two patient groups were generated: those in who the external fixator was entirely removed prior to sterile preparation and draping (“non-prepped”), and those in who at least one component of the external fixator was sterilely prepared into the surgical field (“prepped”). Patients with fixator components “prepped” were sub-categorized: (1) pins only were “prepped” after other components were removed followed by flash sterilization and re-application of construct or (2) the entirety of the external fixator was sterilely prepped into the surgical field.

The primary outcome of this study was the rate of post-operative stiffness defined as the requirement of a return trip to the operating room for knee manipulation under anesthesia. All data were stored and analyzed using the Statistical Package for Social Sciences (SPSS) (version 27; IBM, Armonk, New York). Demographic and outcome variables were assessed using Student’s T-test for continuous variables and Pearson’s Chi-square Test for categorical variables. Sub-analyses for the “prepped” groups applied Pearson’s Chi-square test. Variables with p-value < 0.200 on univariate analysis for the primary outcome were included in regression models to determine potential confounders. The relative risk of postoperative stiffness for binary predictor variables was calculated using odds ratios generated by multiple binary logistic regression analysis described by Zhang, et al [11]. P-values ≤ 0.05 were considered statistically significant.

## Results

A total of 283 patients underwent staged management for high-energy tibial plateau fractures across the two locations during this study period. Thirteen patients were excluded for the presence of an ipsilateral tibial fracture, 3 for definitive treatment using a hexapod frame, 14 for inadequate follow-up, and 9 for insufficient records. In part, 244 patients met inclusion for the final analysis.

One hundred sixty-two patients (66.4%) were included in the “prepped” group, while 82 patients (33.6%) were included in the “non-prepped” group. Seventy-two (44.4%) patients in the “prepped” group had pins sterilely prepared only followed by flash sterilization and reapplication of the construct (Subgroup 1), and 90 (55.6%) patients had the entire fixator sterilely prepared. No differences were noted between the “prepped” and “non-prepped” groups with respect to age, gender, BMI, open fractures, compartment syndrome diagnosis, or OTA classification. The “non-prepped” group remained in the external fixator for more days on average (15.9 vs. 12.3 days, p < 0.001). The average follow-up was 14.6 months (3 months to 60 months) (Table 1).

**Table 1:** Patient Demographics

	Total (N=244)	Not prepped (N=82)	Prepped (N=162)	p-value
Age (years)	50.1 ± 14.4	50.8 ± 15.7	49.7 ± 13.7	0.588
Gender				0.709
Male	135 (55.3%)	44 (53.7%)	91 (56.2%)	
Female	109 (44.7%)	38 (46.3%)	71 (43.8%)	
BMI (kg/m^2^)	29.4 ± 6.6	29.0 ± 6.4	29.6 ± 6.7	0.447
Open Fracture				0.858
No	219 (89.8%)	74 (90.2%)	145 (89.5%)	
Yes	25 (10.2%)	8 (9.8%)	17 (10.5%)	
Compartment Syndrome				0.128
No	219 (89.8%)	77 (93.9%)	142 (87.7%)	
Yes	25 (10.2%)	5 (6.1%)	20 (12.3%)	
OTA Classification				0.171
41B3	39 (16.0%)	17 (20.7%)	22 (13.6%)	
41C1	60 (24.6%)	16 (19.5%)	44 (27.2%)	
41C2	68 (27.9%)	19 (23.2%)	49 (30.2%)	
41C3	77 (31.6%)	30 (36.6%)	47 (29.0%)	
Time in External Fixator (days)	13.5 ± 7.4	15.9 ± 8.4	12.3 ± 6.5	< 0.001
Operative Time (mins)	186.3 ± 79.9	221.9 ± 96.7	168.2 ± 63.0	< 0.001
Number of Incisions				0.639
1	105 (43.0%)	37 (45.1%)	68 (42%)	
2	139 (57.0%)	45 (54.9%)	94 (58.0%)	

The rate of postoperative stiffness across all groups was 10.7%. Patients in the non-prepped group had an increased risk of stiffness postoperatively (18.3% non-prepped versus 6.8% prepped; p = 0.006). There was no difference in postoperative stiffness after subgroup stratification based on sterile preparation of pins only or the entire fixator construct (6.9% pins only versus 6.7% entire construct; p = 0.998). No other variables had p < 0.200 for postoperative stiffness, including the number of days spent in the fixator and operative time. Multivariate binary regression included all variables in table 1 with p < 0.200 to control for differences between the “prepped” and “non-prepped” groups. Preparation status maintained its significance after controlling for these differences. The relative risk for postoperative stiffness associated with not prepping in the fixator was 2.28 (95% CI 1.08-4.14; absolute risk reduction 11.5%; p = 0.032) (Table 2).

**Table 2:** Multivariate Binary Logistic Regression Results of multivariate binary logistic regression for postoperative stiffness including preparation status and confounding variables that differed between the "prepped" and "non-prepped" groups. All included variables had a p < 0.2 on univariate analysis.

Operative Time	B	Odds Ratio	95% CI	p-value
External Fixator Not Prepped	-.988	2.687	1.088, 6.635	0.032
Compartment Syndrome	-.038	0.963	0.205, 4.522	0.963
OTA Classification	-.249	0.779	0.515, 1.178	0.779
Days in External Fixator	-.040	0.961	0.901, 1.025	0.961
Operative Time	.005	1.005	1.000, 1.010	0.073
Constant	-.106	0.900	--	0.945

## Discussion

Postoperative knee stiffness following surgical management of tibial plateau fractures has been reported in occur in a range of 3-18.5% of cases [1,6,7,8,12]. Furthermore, 4-12% of cases have been reported to go on to require secondary intervention for postoperative stiffness [7,10,13]. Stiffness has been more frequently observed after high-energy fractures [6,7,8,12]. Kugelman et al reported that 70% (7.3%) of patients necessitating secondary intervention sustained a bicondylar fracture [10]. Barei et al reported 7.2% of bicondylar tibial plateau fractures required a secondary procedure for limited postoperative range of motion [8]. In another study, Reahl et al reported 12% of all tibial plateau fractures received secondary intervention for post-operative stiffness [9]. This is consistent with the overall rate of postoperative stiffness requiring a secondary procedure of 10.7% found in this study.

Previous literature has demonstrated that open fractures, fracture dislocations, associated tibial eminence fractures, and polytraumatized patients are independent short-term predictors of a decreased postoperative range of motion after surgical management of high-energy tibial plateau fractures [10]. Likewise, increasing patient age, surgical site infection, concomitant non-lower extremity injuries, and non-white ethnicity have been identified as independent long-term predictors of a decreased postoperative range of motion [9,10]. Multiple recent studies have also cited both use of an external fixator and length of time in the external fixator as risk factors for increased post-operative stiffness [6,9,10]. Haller et al demonstrated that the use of temporary external fixation increased the odds of developing postoperative knee stiffness for all tibial plateau fractures in their cohort [6]. After controlling for fracture type, Kugelman et al found temporary external fixation to be an independent risk factor for knee stiffness at short-term follow-up. They demonstrated no correlation with the length of time in the external fixator on multivariate analysis [10]. In contrast, Haller et al demonstrated that each day spent in external fixation conferred an additional 10% increased risk of requiring secondary intervention for postoperative stiffness [6]. Similarly, Reahl et al reported a 7% increase for each day in an external fixator [9]. Based on the results of the above studies, it is apparent that the use of temporizing external fixation likely increases the risk of clinically significant postoperative stiffness after tibial plateau fractures. However, these studies included all tibial plateau fractures and not just those that are typically considered high-energy. Based on this data it is difficult to determine the true cause of postoperative stiffness, as high-energy injuries inherently present with more damage to the bone and surrounding soft tissues, often require more extensive surgical dissection, and are typically associated with longer durations of rehabilitation that may delay range of motion postoperatively.

Though staged management is frequently used for high-energy fractures of the tibial plateau, the question of what to do with the external fixator has not been answered [1,2]. In the present study, the rate of post-operative stiffness was shown to be more than 2.25 times greater in the non-prepped group compared to the prepped group (18.3% non-prepped versus 6.8% prepped), independent of other independent predictors of postoperative stiffness, including time in the external fixator and operative time. This study suggests that the removal of the external fixator may be associated with increased postoperative stiffness independent of time in the external fixator. On a molecular level, arthrofibrosis or stiffness has been explained by a combination of genetic factors and over-expression of inflammatory cytokines, specifically transforming growth factor-β (TGF-β) and platelet-derived growth factor [14,15]. This leads to fibroblast and extracellular matrix proliferation, protease inhibition, and collagen formation. Furthermore, a genetic sensitivity to a hyperactive inflammatory system can exacerbate scare formation and tissue regression [16]. We hypothesize that the transient loss of reduction associated with external fixator removal may lead to additional inflammatory insult and articular trauma. Interestingly, our data did not demonstrate longer duration in the external fixator to be associated with increased rates of intervention for postoperative stiffness (13.6 days for those with post-operative stiffness versus 13.1 days for those without, p = 0.75), which contrasts with the findings of prior studies [6,9,10]. The present study also found that increased fracture severity based on AO/OTA classification did not associate with rates of postoperative stiffness, similar to the findings of Haller et al [6].

This study has several limitations. Because of its retrospective nature, the decision to maintain the external fixator was based on the assessment of the managing surgeon. This could have introduced some inherent bias that was not captured in the analyses. A radiographic assessment of reduction quality was not completed in this study. Expansion on this outcome with both assessment of radiographic reduction after external fixator placement and time to radiographic union in each group as it compares to postoperative stiffness may help elucidate reasons for the findings presented above and is another target for future study. Physical therapy documentation was not uniformly accessible to determine functional progression and patient adherence as it related to stiffness. Finally, pre- and post-secondary intervention range of motion was not uniformly monitored to determine effectiveness. However, by defining postoperative stiffness as the need for a secondary manipulation procedure, this study likely was able to capture those patients who did have clinically significant stiffness after their initial fixation. 

## Conclusions

This study demonstrates that maintaining an external fixator during the definitive treatment of high-energy tibial plateau fractures with staged management is associated with a decreased rate of postoperative stiffness requiring secondary intervention when compared to complete construct removal prior to surgery. No other reported variables were found to be associated with an increased risk of subsequent stiffness in this study. Orthopaedic surgeons who treat these injuries should be aware of the risk factors as it relates to an impaired range of motion and may consider preparing an external fixator in the surgical field as a low-risk and readily available reduction tool to aid to achieve optimal postoperative function. 
